# Social media users' free labor in Iran: Influencers, ethical conduct and labor exploitation

**DOI:** 10.3389/fsoc.2022.1006146

**Published:** 2022-11-10

**Authors:** Leilasadat Mirghaderi

**Affiliations:** Department of Humanities, Illinois Institute of Technology, Chicago, IL, United States

**Keywords:** audience labor, exploitation, ethical conduct, influencer culture, political economy

## Abstract

As social media sites are penetrating our daily lives in an ever-increasing manner, there is a need to revisit and reexplore the theoretical concepts that have gone through paradigm shifts due to the influence of these platforms. In this regard, audience labor theory, which was originally conceptualized in the context of mass media, needs to be reexamined as the divide between production and consumption is getting narrower. Users are no longer passive consumers since social media sites have reduced the cost of production and resulted in the advent of the term “prosumption.” In such a case, as production involves performing work and results in surplus-value, it needs to be investigated whether users are being exploited for the free work they provide on these platforms. From the several identified forms of digital labor, I will focus on the concept of audience labor. To this end I will focus on identifying labor strategies that Iranian Instagram influencers employ; these strategies involve exploiting their followers to perform tasks that produce fame and visibility as well as monetary gains but leave the users uncompensated for the work they have performed. By conducting content analysis of the 2,130 stories created by 71 Iranian Instagram influencers, this study will identify the strategies that these influencers use to exploit their followers.

## Introduction

Social media sites have become an inseparable part of our daily lives, especially with the ubiquitous access to the internet and the mobility that smartphones provide. Over recent years, social media sites have caused several paradigm shifts in theoretical concepts. One particular concept that is heavily impacted by digital technologies is the labor market and the shift of labor markets to the internet. The internet has created different types of labor with different types of production and compensation (Nakamura, 2015). Furthermore, different internet platforms have reduced the cost of content creation; initially, this was done through blogs and then later through social media sites.

The reduction in the cost of production enabled the users to no longer be passive consumers and to be able to simultaneously produce and consume content [hence the word “prosumption” was coined (Lowgren and Reimer, 2013)]. Furthermore, without spatial and temporal constraints, content on the internet can virtually reach audiences of unlimited size. The collection of these characteristics of the internet platforms has resulted in several paradigm shifts. In the context of digital media, terms such as free labor (Terranova, 2012), audience labor (Fisher, 2015), and fame labor (Mavroudis, 2018) all refer to how social media users create surplus value for these platforms by their willing participation without appropriate forms of financial compensation.

Among the different types of digital labor and their associated challenges, the focus of this paper is the free labor of social media sites users (Terranova, 2012; Nakamura, 2015). In this context, free labor refers to the voluntary and unwaged activities that social media users provide for influencers. In the context of audience labor, this separation of the social media users from the profit that is generated from the commodification of their activities is referred to as exploitation (Nixon, 2020). Through the lens of audience labor, I investigated the free labor users provide by following influencers, which is one a wide range of users' activities that result in free labor. Social media influencers are ordinary internet users who become famous by attracting a large number of followers or subscribers to their social media page and share advertorial posts on their social media pages for monetary gains (Abidin, 2015). With the large number of followers that social media influencers possess, they attract brands as new channels for advertising. Furthermore, as we will see in this paper, advertorial content by the influencers is not the only method influencers use to exploit the free labor from their audience as there are non-material aspects as well.

Users of social media platforms spend a lot of time every day visiting, following, or liking influencers' accounts and their content. These different forms of attention can be viewed as virtual currencies in this “attention economy” (Drenten et al., 2020). Among every few non-advertorial posts (content can be satirical, everyday life experiences, or fashion and makeup-related guidelines), there are posts in which the influencer promotes a product or service and gets paid in return from the sponsors. As Mavroudis (2018) asserts, having a large number of followers is a necessity for influencers to get paid as a result of their posted ads, and the number of followers has a direct impact on how much influencers get paid. Based on performing a qualitative content analysis of the content of Iranian social media influencers, I argue that users are providing free labor, while influencers are getting paid for it and brands are benefiting from the reduced cost of advertising. Specifically, I identify the labor strategies Iranian Instagram influencers are employing to exploit their followers to get them to perform tasks that produce fame and visibility as well as monetary gains, while the followers are left uncompensated for their work.

In this article, I will start by providing a brief historical background about the relation between technology, politics, and society in Iran. This study is focused on Instagram and Iranian users of this platform. The reason for this selection is that currently, Instagram has a much higher amount of active Iranian users than Facebook and Twitter (Azali, 2017), with an estimated total of 20 million users. In the literature review section, I will provide a background about the history of digital media in Iran as well as Western theoretical concepts that applies to this research such as free labor and audience labor in digital media. In the methodology section, I will explain the research method that I employed in this research and present the collected data. Then, I will share and explain the results of the analysis. Finally, I will conclude this paper.

## Literature review

### History of the internet and digital media in Iran

Among the Middle Eastern and North African countries (MENA), Iran can be regarded as a frontrunner in acquiring new information and communication technologies (ICTs) technologies. However, contrary to the common belief, the Iranian government viewed digital technological advancements as the key to boosting its economy, promoting its image as a modern state, and developing strategic leverage over its competitors in the region. Moreover, Iranian citizens have shown enthusiasm for adopting new technologies. According to Falasiri and Ghanavizi (2015), this enthusiasm and appreciation may be associated with the relatively high literacy rate (77%), and high education level in Iran. For instance, in the early 2000s, Iran had one of the fastest internet usage growth rates in the world (Falasiri and Ghanavizi, 2015), and in 2012, Iran had the largest number of internet users in the Middle East (Nasirzadeh, 2015).

In the contemporary history of Iran, the Islamic Revolution of 1979 is a significant milestone, in which the Pahlavi Dynasty was overthrown and replaced by the Islamic Republic. The feud toward the West after 1979 led to the isolation of Iran on the international. Accompanied by 8 years of war with Iraq (1980–1988) right after the revolution, the existing technological infrastructure was destroyed, and Iran was deprived of access to technological advancement in the West due to sanctions that took a toll on its economy (Michaelsen, 2009).

In such a predicament, the internet seemed to be the perfect solution for a global reconnection with the world and compensating for the losses during the previous decade. Contrary to the expectation, the Iranian government promoted the internet as a platform for scientific communication and academic purpose, without any restrictions (Rahimi, 2003). However, like most other places in the world, the usage of the internet among Iranians spread soon after it became available, going beyond any specific academic standing, age, class, gender, or religious belief. That is why in just a few years, the internet user base in Iran expanded from the academic community to the whole public.

Internet was one of the technological advancements that was adopted in Iran soon after it became available. Iran was the second country in the Middle East to gain access to the internet (Rahimi, 2003). The first email in Iran was sent in January 1993. In only a little more than a decade, by the year 2005, the number of internet users in Iran reached five million which was one of the fastest growth rates in the world (Falasiri and Ghanavizi, 2015). In its 2017 report, the Telecommunication Company of Iran (TCI) stated that there are 47 million internet users in 11,000 cities and villages of Iran (TCI, 2017), a country with a total population of 81 million. However, the path to acquiring new technologies in Iran has not been very smooth due to being isolated in a world that is becoming more and more globalized. Regardless, as savvy tech users, Iranian internet users adopted different internet platforms such as blogs and then social media sites soon after they became available.

Blogs were one of the earlier forms of communication channels on the internet that Iranians employed. Iranians had already realized the universal and egalitarian nature of the internet and started to use it as a new channel for self-expression. At the time, the anonymity that blogs could provide was well received by the Iranian internet users since they could share their thoughts and opinions freely without revealing their identity (Falasiri and Ghanavizi, 2015). As one of the early stages of self-expression on the internet that provided anonymity by the use of pseudonyms, blogs were well received by the Iranian internet users. However, the advent of social media sites changed self-expression in Iran once again.

When social media sites such as Facebook and Twitter were introduced, new platforms with several new and different features became available for Iranian internet users. Social media sites allowed users to easily connect with each other, a feature that was not available in blogs. Also, Facebook provided the ability to create groups so that like-minded people can connect with each other, and sometimes these online communications could result in offline gatherings (Gheytanchi, 2015). However, when these two platforms played a facilitating role in the protests against the results of the presidential election of 2009, they became the subject of restriction by the government. Since 2009, the government has been perceiving both platforms as political threats and blocked all Iranian internet users from accessing these websites.

Facebook and Twitter were permanently blocked by the Iranian government after they were used by the Iranian users for organizing protests against the results of the 2009 presidential election, and they still remain inaccessible after almost a decade (Kargar and Rauchfleisch, 2019). Currently, Iranians have to use unreliable tools such as proxies and virtual private networks (VPNs) to access Facebook and Twitter, which usually result in connectivity issues. Over the past decade, the usage of Facebook and Twitter among Iranian citizens has had a downward trend. Currently, Instagram is the only major accessible social media platform in Iran. The ease of access to Instagram and its more appealing visual features have led to the gradual migration of Iranian social media users from Facebook and Twitter to this platform. Currently, the number of Iranian active users on Instagram is remarkably higher than Facebook or Twitter (Azali, 2017). In 2018, Iran ranked 7th in the world for the number of Instagram users (Financial Tribune, 2018).

A relatively new trend that did not exist before the popularity of Instagram in Iran is the emergence of influencers and their advertising strategies. The impact of influencer culture in Iran is to a level that government officials have estimated that restricting access to Instagram can cause a loss of income for up to one million Iranians (BBC Persian, 2021). In the following sections, I will first provide background regarding influencer culture and advertising based on Western research and then use content analysis to investigate and identify the strategies that Iranian influencers are utilizing in this context.

### Influencer advertising and free labor on social media in western countries

Instafame, Instagram micro-celebrity, and Instagram influencer are all terms that are related to the condition in which a user has a large number of followers through sharing inspirational photos and videos on their Instagram page (Marwick, 2015; Evans et al., 2017). Micro-celebrities exist on many social media platforms, however, their presence on Instagram is different from other platforms since individuals use photos and videos to describe themselves and their experience rather than plain text (Marwick, 2015).

Alongside the features that enable users to communicate with each other, Instagram can be used as a potent marketing tool by brands and businesses to connect with their target audience (Ewers, 2017). Social media sites, and Instagram, in particular, were able to reduce the cost of advertising for brands and businesses. While brands can have an online presence by their dedicated accounts, influencer marketing provides a novel and indirect approach, in contrast to the traditional direct marketing approaches. With influencer marketing, a brand can work with influencers that have a considerable number of followers that match the brand's target group (Ewers, 2017). A study by Jarrar et al. (2020) suggests that influencer marketing can result in more sales than brand-sponsored posts. While there is a need for more research on the effectiveness of influencer marketing compared with sponsored posts, it is already established that brands are using influencer marketing to improve their image and sales. Advertising through influencers is an important method for brands and business owners to promote their product and services since the followers usually trust influencers, and they also mimic the style of influencers by using the products that the influencers use (Abidin, 2016). Furthermore, followers use the same tags that influencers use and they tend to repost the contents that influencers share (Abidin, 2016). Therefore, followers of the influencers are exposed to a large number of advertisements that brands broadcast through influencers. Followers might even share these advertisements with their connections without being paid which is very beneficial for business owners who want to promote their products and services (Abidin, 2016). In this context, a concept that needs attention is the audience labor.

Projecting the theoretical framework of labor, which was developed for the nineteenth-century factories, to the leisure activities in the twenty-first- century was a challenging task. Researchers were confronted with challenging questions such as, “How can we talk about a leisure activity as labor? And how can we talk about exploitation when people voluntarily watch broadcast television for free?” (Fisher, 2015). Originally conceptualized for the mass media, proponents of this theory would argue that “economically speaking mass communication was not about audience consuming free content—produced by media corporations—but, in fact, about the selling of audience attention to advertisers” (Fisher, 2015). On the other hand, the critiques of audience labor would target the passive role of audiences with the mass media. However, with the advent of social media, users can no longer be perceived as passive agents. Compared with the users of mass media, social media users are more active, engaged, and creative, and are able to create content without being compensated for their time and energy. Moreover, they can also share the content created by other users with their network and increase the original content creator's viewership. Such active role of social media users on these platforms, from the aspects of content creation and propagation, as well as the economy of these platforms, justify the revisiting of concepts such as audience labor. As Terranova (2012) states, there is a continuity and a break between mass media and the new media. The continuity is in how both media platforms put users at the center of the political economy of the media and the difference lies in the ways that users produce surplus value.

Content is no longer just created by professional producers, and users have gained a significant role in content creation due to the decreased cost of using content creation tools. While it appears that users are freely accessing platforms, users are no longer just consumers of content: they also produce it as well. Therefore, terms such as “prosumption” (production and consumption) or “produsage” (production and usage) were introduced (Lowgren and Reimer, 2013). This immaterial, voluntary, and free labor of users has resulted in the need for a critical political economy approach that does not exclude the users from the economic analysis but takes them into account as integral and crucial parts of the functioning of social media platforms economy (Fisher, 2015). Similarly, (Paakkari et al., 2019) suggest that the internet research should put more emphasis on the opinion of the users. One of the ways that critical political economy sheds light on the importance of users is the explanation of how users are alienated (right to have control) and exploited (right to have profit) from the content they create (Fisher, 2015). However, scholars such as Jenkins et al. (2015) do not consider the these platforms to be exploitative due to their empowering characteristic in the participatory culture.

Free labor of users on social media sites can be categorized into two groups. The first group consists of the voluntarily content creation and productive activity of users that directly transforms into profit for the private companies that own these platforms (Cohen, 2013). For this category, the extreme case would be the content created by the influencers. As Mavroudis (2018) states, influencers usually feel the pressure to constantly participate, create content, and collect likes and comments. The second group results from the participation of fans in following influencers, liking and commenting on their content (both non-advertorial and advertorial) which results in financial profits for influencers and consequently, the private companies. As there has been extensive research regarding the former category, and in this paper, I focused on the latter. Regardless of the type of free labor on these platforms, the information of users on social media sites is subject to alienation and exploitation.

Furthermore, according to Andrejevic (2011), information created on social media sites can either be intentional or unintentional. Intentional information is generated by the intentional actions of users such as sharing user-generated content. Unintentional information is the data that users produce unintentionally while they are performing other tasks. For instance, while making a purchase online or following accounts that match our identity, we are revealing user-behavior data that has value to commercial platforms, and we have little to no control over the generation of this kind of information or how it is being used (Andrejevic, 2011).

The information that users provide facilitates commercial exploitation by private companies. In this regard, Fisher (2015) explains how users are both extensively and intensively exploited. Extensive exploitation refers to the arrangements that increase work time and in the case of social media sites, users are exposed to more advertisements, and consequently, they spend more time with advertisements. Intensive exploitation refers to producing more in less time; in the case of social media sites, intensive exploitation refers to targeting a specific population and presenting shorter commercials while maintaining the total exposure time (Fisher, 2015). In the case of this research, stories would be a great example of both extensive and intensive exploitation of Instagram users.

Considering the background regarding influencer advertising and the current influencer and creator culture in Iran, I have identified the following research question: What labor strategies do Iranian Instagram influencers employ by exploiting their followers to perform tasks that produce fame and visibility as well as monetary gains?

## Methodology

As it was stated in the literature review section, due to the current conditions in Iran, Instagram has the highest number of active users in Iran compared to other major social media platforms such as Facebook and Twitter. As a relatively new social media platform that gives freedom to the users as to the type of content they would like to generate (such as sharing daily life or making satirical, educational, political, and promotional content), Instagram has become the arena for users with different sets of talents who are seeking fame. In such an environment, many influencers are rising and becoming popular by employing techniques to attract more followers, likes, and comments, and other methods that I will explain in more detail. Instagram influencers can turn their fame into financial profit without compensating the users who provide the attention influencers require. By analyzing the Instagram stories of the Iranian Instagram influencers, based on performing content analysis, I extract themes of how these influencers take advantage of users as a form of free labor to gain more fame and profits for themselves.

In this paper, I employed a qualitative content analysis to address how Iranian internet users are providing free labor on social media sites for influencers and brands. Regarding qualitative research, Dubljević et al. (2021) assert that it is a “social science methodology that collects non-numeric, value-laden data. Although qualitative research is not generally known in computer science, outside of a few specialties, it is a useful approach for addressing the ethical and social implications of technology.”

Content analysis, as an approach for textual analysis, facilitates studying mass-mediated and public messages by counting “characteristics of messages embedded in public and mediated texts” (Frey et al., 2000). The aim of this method is to determine the recurrent topics that were dominant in the dataset. Such a research method is useful for studying the content on the internet to systematically reduce a large amount of data. Therefore, with content analysis, audience labor as a concept can be operationalized by a list of activities that are listed in the results. The first step was to gather the data. After familiarization with the data, the coding process starts which involves classifying the data based on pre-defined categories identified by the researcher.

## Data collection and analysis

First, I compiled and ordered a list of 200 prominent Iranian Instagram influencers, based on the number of their followers. Number of followers was chosen as the criteria for ordering and selecting these followers as followers count significantly changes the income of influencers. According to Mavroudis (2018), the same brand offer for an influencer with 400,000 followers can be a hundred times higher than an influencer with 27,000 followers. Then, I selected the first 71 influencers for this study, with the first influencer having five million and one hundred thousand followers, and the last influencer having more than twelve thousand followers, at the time of this study. Based on personal observation, influencers with follower count of more than 10,000 are able to attract businesses and engage in sharing ads. It should be noted that I was born and raised in Iran, with Persian as my native language, which make me equipped with the means that facilitate this study. Also, I had active participation in this community since 2013, and as a researcher since 2019. Furthermore, the reason for selecting the first 71 influencers is that considering the limited time of this research, this number provides enough data to result in saturation. In this context, saturation can be identified as when adding new strata to the corpus makes minimal changes to the representation (Bauer and Aarts, 2000). Each of these influencers focuses on a certain type of content generation, such as sharing daily life, makeup lessons, fashion styling, music production, satirical content production, and food blogging.

After familiarizing myself with the influencers and their content created, I observed that most of the advertising is performed in stories, rather than posts. Instagram stories, unlike posts, are not permanent and disappear after 24-h and that may be the reason why posts are used for original content by the influencers and stories are used for advertising. However, there is a need for further research on this matter which is beyond the scope of this paper. I studied 30 Instagram stories (one of the features of the Instagram platform that will remain visible only for 24 h) from each influencer. This study was performed from January to April 2021.

In total, I analyzed 2,130 Instagram stories (30 stories from each influencer) and the results of this analysis are presented in the next section. This number of stories were chosen considering the timeline of the project. Furthermore, upon seeing each story, I coded that story based on a set of pre-defined categories that I had previously identified. Based on the analysis of the Instagram stories, I extracted two themes and six strategies that are explained in detail in the following section.

## Results

Based on the analysis of the stories, I extracted six strategies that Iranian Instagram influencers use to exploit their audiences. Furthermore, these strategies fall into two themes of non-material and material capital (see Table 1). Non-material capital encompasses the strategies that are used to increase fame and visibility. In other words, non-materials capital is associated with strategies that influencers employ to increase their exposure by benefiting from the free labor of their followers. On the contrary, the material capital theme includes strategies that directly result in monetary gains, such as advertising. While some of these strategies are prevalent and supported with high frequency, some others might have low frequency but illustrate a major issue. That is why in general, qualitative research is “less interested in the issue of representativeness than in the content, organization and functions of texts” (Gill, 2000). Furthermore, as it can be seen from the frequency of the strategies, the summation of these exploitative strategies is less than the total number of studied stories, since not every story is exploitative.

**Table 1:** Extracted themes and strategies.

**Themes**	**Strategies**	**Frequency**
Non-material Capital	Relegate content creation to manufacture account visibility	554
	Emotional blackmail to solicit engagement	148
	Nurture collaborative inter-influencers strategies	144
	Weaponizing followers	23
Material Capital	Native Advertising	94
	Clickbait	71

For the non-material capital theme, four strategies were identified based on the analysis of the data. First, influencers relegate the task of content creation to the followers themselves. Second, they perform emotional blackmailing to solicit engagement. Third, influencers use their followers to boost their own inter-influencers relations. Finally, in contrast to the previous strategy, influencers might attack a rival influencer using their followers. For the material capital theme, two strategies were identified. Influencers use native advertising which is a way of sharing an ad in the disguise of regular non-advertisement content. Finally, to increase the traffic to the account or website they are advertising, influencers might use deceiving headlines, which is similar to the concept of clickbait.

For the first strategy of the non-material capital theme, influencers use question boxes and ask trivial questions such as “what's up?” Next, they keep sharing the answers of followers in the next couple of stories. Being active and producing a lot of content is a key for influencers in order to keep their followers and also attract sponsors. In this regard, Mavroudis (2018) asserts that influencers use stories to keep followers engaged for the time being, when they do not have good enough content to post. What can be perceived here is that influencers are relegating the task of creating stories to the followers.

Second, influencers try to boost their own page in a non-organic manner by putting an emotional burden on the followers. In the context of social media sites, organic growth refers to the case that users only employ tools available on social media sites to increase their followers and exposure, and it is contrasted with inorganic growth which is the use of promotions and automated tools (Bellavista et al., 2019). For instance, if their latest content does not attract enough attention, in terms of likes and comments, influencers might tell how sad they are because of not receiving enough attention or threaten their followers that they will not create new content until their last post receives enough attention. In the current “attention economy” of social media sites, likes and comments are like virtual currency for influencers, and they lose their foothold if they cannot attract enough attention (Marwick, 2015; Drenten et al., 2020). Such attention should be gathered organically, and emotional blackmailing of followers may not be an appropriate way of gaining attention.

The third strategy is related to shoutouts. According to Drenten et al. (2020), “shoutouts are intended to show support and give exposure to other users and can substantially increase a user's followers”. Influencers ask followers to visit the pages of their friends and families. Through using the social capital of strong ties, new influencers with a limited number of followers can boost their page in a non-organic way. Finally, the fourth strategy is the opposite of the third one. In this strategy, when influencers have some personal issues with a person (such as their rivals) or a company (such as having a bad experience with a product or service), they use their influence over their follower and weaponize them to damage the online presence of that person or company. Influencers ask their followers as a favor to either report the page they announce or put inappropriate comments on the Instagram page of the individual they want to damage.

For the material capital theme, using the same strategy of sharing stories and question boxes, influencers ask questions that seem to be undirected, but they actually are planning to share an advertisement that is related to the question. This is similar to native advertising which is sharing an advertisement when it is being presented as regular content. Other scholars such as Schauster et al. (2016) have raised ethical concerns regarding native advertising. In this strategy, question boxes guarantee interaction from followers and gather eyeballs. When enough attention is gained, influencers share an advertisement, disguised as regular non-advertisement content. For instance, an influencer might discuss the topic of the new year holiday, which is usually universally related to weight gain, and recommend a nutritionist afterward. For the last strategy, influencers use deceptive headlines in their stories when they are tagging another page. Using such headlines may tempt followers and direct traffic to the tagged accounts or websites. This strategy is similar to using clickbait, and influencers use highly exaggerated descriptions to direct their followers to another page.

From the above themes, it can be seen that Iranian Instagram influencers might be taking advantage of the enthusiasm of their followers and use their power and influence to exploit users for their own material and non-material gains. Considering that Instagram is the only major accessible social media site in Iran, Iranian internet users have gained a passion for this platform and much of their online activity is limited to Instagram. This dependability opens room for exploitation, and as it is illustrated in this research, influencers may use this to their own benefit.

## Conclusion and future work

In summary, I explored the strategies Iranian Instagram influencers use to exploit their followers and benefit from the free labor that their followers provide. In this regard, I employed audience labor as a theoretical lens to investigate my research question. By performing qualitative content analysis of 2,130 Instagram stories from 71 Iranian Instagram influencers, I extracted themes and strategies that influencers use to exploit their followers. Although users are voluntarily going through this type of labor and enjoy their activities on Instagram, they are spending time and energy and may not be aware of the importance and value of their labor. Therefore, this research aims to raise awareness about such methods of labor exploitation and have a preventative role in reducing the need for after-the-fact actions. While the aim of this study is to raise awareness by employing a qualitative approach to identify the potential issues, there is a need for quantitative approaches in future studies to take this study a step further and support the suggested ethical guidelines in this paper.

## Data availability statement

The datasets presented in this article are not readily available because the datasets analyzed for this study are not publicly available due to privacy or ethical restrictions. Requests to access the datasets should be directed to lmirghaderi@hawk.iit.edu.

## Author contributions

LM: conception and design of the work, acquisition, analysis, or interpretation of data, and drafting the work or revising it critically for important intellectual content.

## Conflict of interest

The author declares that the research was conducted in the absence of any commercial or financial relationships that could be construed as a potential conflict of interest.

## Publisher's note

All claims expressed in this article are solely those of the authors and do not necessarily represent those of their affiliated organizations, or those of the publisher, the editors and the reviewers. Any product that may be evaluated in this article, or claim that may be made by its manufacturer, is not guaranteed or endorsed by the publisher.
